# Antifreezing and Stretchable Organohydrogels as Soft Actuators

**DOI:** 10.34133/2019/2384347

**Published:** 2019-12-13

**Authors:** Yukun Jian, Baoyi Wu, Xiaoxia Le, Yun Liang, Yuchong Zhang, Dachuan Zhang, Ling Zhang, Wei Lu, Jiawei Zhang, Tao Chen

**Affiliations:** ^1^Key Laboratory of Marine Materials and Related Technologies, Zhejiang Key Laboratory of Marine Materials and Protective Technologies, Ningbo Institute of Material Technology and Engineering, Chinese Academy of Sciences, Ningbo 315201, China; ^2^University of Chinese Academy of Sciences, 19A Yuquan Road, Beijing 100049, China

## Abstract

Inspired by the freezing tolerance performances found in living creatures, an effect approach is presented to develop novel antifreezing polymeric organohydrogel actuators. Through construction of a bilayer hydrogel including a nonresponsive polyacrylamide (PAAm) layer and a pH-responsive polyacrylic acid (PAA) layer in the presence of a mixed solvent of water and glycerol, organohydrogel actuators that could produce various shape deformations at subzero temperatures have been achieved, and the actuating speed could be tuned by adjusting the temperature and the ratio between glycerol and water. Moreover, a series of application demonstrations including a weightlifting robot, artificial valve, and robotic arm have been displayed. In addition, by introducing the ionic compound KI into the glycerol-based organogel, flexible conductors that could perform stable sensing performance over a wide range of temperatures from -30°C to 60°C have been developed.

## 1. Introduction

Living creatures have the most ingenious structures and amazing functions in nature, and they have attracted broad interest and inspired the fabrication of various intelligent materials [1–5]. As one of the most promising smart materials, polymeric hydrogel actuators [6–10] could produce reversible shape transformation in response to various external stimuli such as heat [11, 12], pH [13–15], light [16, 17], chemicals [18], and electricity [19, 20], and in addition to the soft and wet properties, they have shown promising applications as biomimetic actuators [21–23], switches [24, 25], softs robots [26, 27], artificial muscles [28], and so on. However, due to their high water content, hydrogel actuators will freeze at subzero temperatures and dehydrate in air at a relatively high temperature, which will seriously restrict their practical applications. After having evolved for four and a half billion years, some living organisms in nature such as peeper frogs have gained the ability to endure cold environments because their body fluids contain antifreezing agents including salts and alcohols, which prevent them from freezing into ice at extremely cold temperatures. Actually, salts such as CaCl_2_ have been widely used to prevent the formation of ice on the road, and alcohols such as glycerol are effective antifreeze agents in industry.

Inspired by the antifreezing behaviors found in nature [29], recently, a few efforts have been devoted to render hydrogel with freezing tolerance abilities. For example, Liu and coworkers have reported the combination of hydrophilic/oleophilic heteronetworks to construct mechanical stable organohydrogels over a wide temperature range [30]. Cryoprotectants such as ethylene glycol, glycerol, propylene carbonate, and CaCl_2_ have been introduced into hydrogels to realize antifreezing capacity [31–33]. In addition, alcohol moieties could be anchored onto polymer chains to prevent the hydrogel from freezing [34]. Taking advantage of the antifreezing behavior, some other interesting hydrogel-based devices such as sensors and batteries could also be operated at subzero temperatures [35–37].

**Figure 1:**
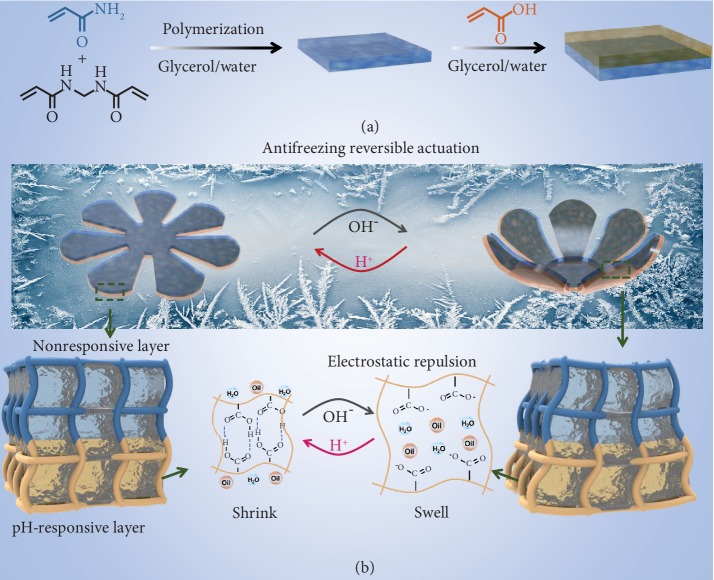
Schematic illustration of the preparation and actuation mechanism of the gel actuator. (a) First, a nonresponsive polyacrylamide (PAAm) layer was prepared by free-radical polymerization, then a pH-responsive polyacrylic acid (PAA) layer was introduced as a deformation layer to achieve a bilayer organohydrogel. (b) The reversible deformation mechanism of the bilayer organohydrogel. The PAA layer can be unprotonated under alkaline conditions, the electrostatic repulsion among the carboxyl groups would cause the PAA layer to swell, and the bilayer organohydrogel would deform as a result of the swelling of the PAA layer.

Considering that the introduction of an antifreezing agent into hydrogels will endow them with a freezing tolerant capacity, it can be anticipated that hydrogel actuators could also generate motions at subzero temperatures using a similar strategy. Herein, we present stretchable organohydrogel actuators with antifreezing performance. First of all, a polyacrylamide (PAAm) organohydrogel with a glycerol-water binary solvent was prepared; the strong hydrogen-bonding interactions between glycerol and water molecules not only prevent the organohydrogel from freezing at low temperature but also hinder water molecules from evaporating in air, giving the gel a long-term durability. Then, a pH-responsive polyacrylic acid layer was combined to build a bilayer organohydrogel actuator; the obtained soft actuators could work well at subzero temperatures and could be applied as a weightlifting robot, artificial valve, and so on (Figure 1). In addition, an ionic compound KI was introduced into the glycerol-based PAAm organogel to achieve a conductive gel sensor that could be used at a wide temperature range from -30°C to 60°C. To integrate antifreezing and sensing abilities, our strategy offers a simple way to expand the working environment of soft actuators and may also provide new ideas in fabricating soft sensors with extreme temperature tolerance.

## 2. Results

### 2.1. Antifreezing and Mechanical Enhancement of the Organohydrogels

**Figure 2:**
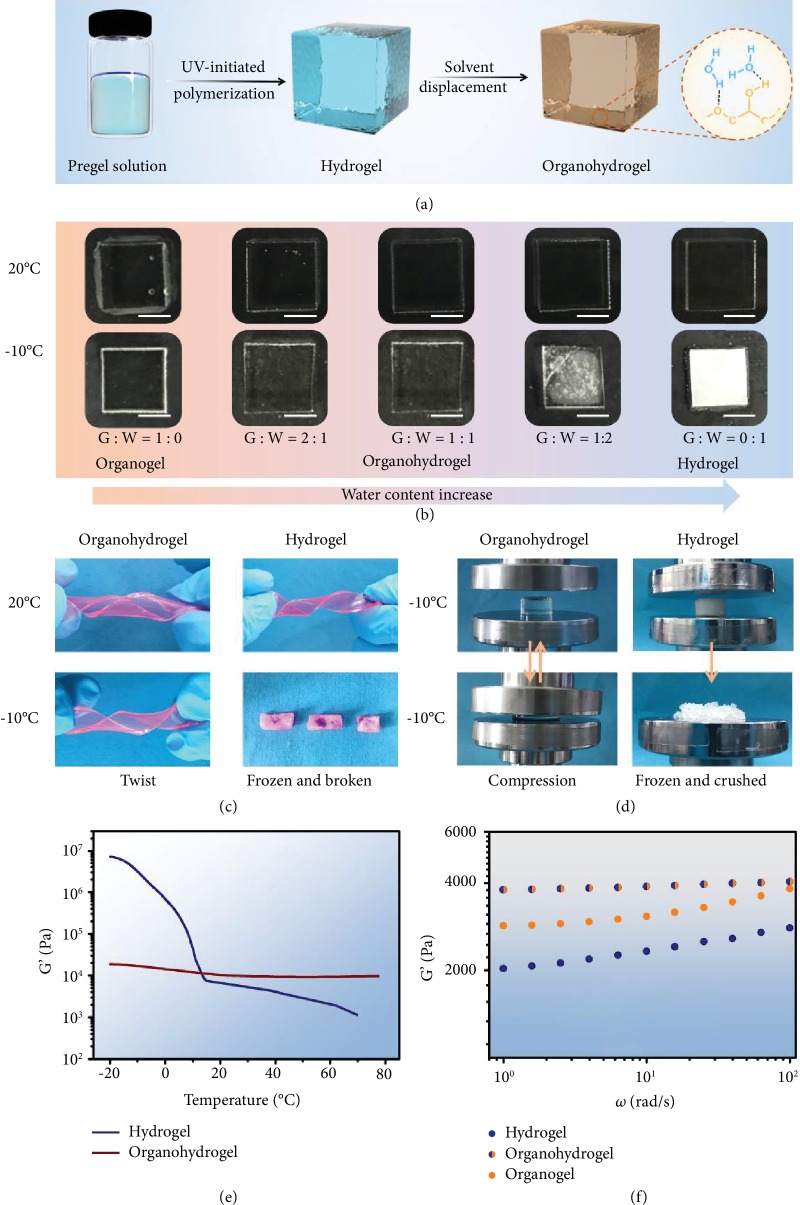
The antifreezing properties of the PAAm organohydrogel. (a) Schematic illustration of the solvent-displacement method. (b) Digital photos of gel blocks with different solvent components at 20°C and -10°C (scale bar: 5 mm). (c) The hydrogel and organohydrogel (glycerol : water = 1 : 1) are twisted at -10°C. (d) Compressive properties of the organohydrogel (glycerol : water = 1 : 1) and hydrogel at -30°C. (e) G′ of the hydrogel and organohydrogel (glycerol : water = 1 : 1) on a temperature sweep in the range of -20°C to 80°C at a constant shear strain of 0.1% and frequency of 10 rad/s. (f) Frequency scanning rheological tests of the organogel, organohydrogel (glycerol : water = 1 : 1), and hydrogel at a constant shear strain of 0.1%.

Glycerol has been applied in invertebrates to prevent the body fluid from freezing at subzero temperatures, and it is also widely used as antifreezing agents in the industrial field. The introduction of glycerol into hydrogels may also render them with an antifreezing capacity. In order to investigate the influence of glycerol on the antifreezing properties of gels, glycerol-based PAAm organohydrogels were prepared with the solvent-displacement method (Figure 2(a)). Briefly, the original PAAm hydrogels were immersed in a water-glycerol binary solvent, in which the ratios of glycerol to water were 1 : 0, 2 : 1, 1 : 1, 1 : 2, and 0 : 1, to generate binary solvent-based organohydrogels. Then, the as-prepared gels were put in a -10°C refrigerator to observe their antifreezing behavior. As illustrated in Figure 2(b), organohydrogels with 50% or more glycerol content are not frozen, ice crystals appear on the gel with 33% glycerol content, and the pure hydrogel is even frozen directly. The results indicate that the introduction of glycerol can effectively reduce the freezing point of the hydrogel. Figures 2(c) and 2(d) and [Supplementary-material supplementary-material-1] illustrate the difference in antifreezing behavior between the two gels. After storage at -10°C for 6 h, the mechanical properties of the organohydrogel are not much different from those of hydrogels at room temperature, showing great elasticity to withstand large deformations including stretching, twisting, and compression. On the contrary, ordinary PAAm hydrogels are frozen at -10°C and could easily be broken into pieces when stretched, twisted, or compressed. To further evaluate the influence of temperature on the elasticity of pure hydrogels and organohydrogels, dynamic mechanical analysis (DMA) was performed and the results are shown in Figure 2(e). When the temperature is increased from -20°C to 10°C, the storage modulus (G′) of the pure hydrogel rapidly reduces from 10^7^ to 10^4^ Pa, which can be attributed to the melting of ice crystals at around 0°C. In the organohydrogel, strong hydrogen bonds are formed among glycerol molecules, water molecules, and the PAAm network, which will hinder the crystallization of water molecules; therefore, the G′ of the organohydrogel is stable at subzero temperatures. Moreover, since the hydrogen bonding between the polymer network and water molecules will become weaker with increasing temperature, the G′ of both the organohydrogel and the hydrogel continues to decrease above 0°C because of dehydration. In addition, the hydrogen-bonding interactions could also enhance the mechanical properties of the organohydrogel. As shown in Figure 2(f), the frequency-sweep rheological tests of gels with different solvent components show that the G′ of the glycerol-based organogel is higher than that of the pure water-based hydrogel and G′ of the two gels is lower than that of the binary solvent-based organohydrogel. It can be assumed that the glycerol-water mixture exhibits stronger hydrogen-bonding interactions with polymer networks than pure glycerol or water, which is consistent with a previous report [38].

The strong hydrogen bonds between glycerol-water and polymer networks not only prevent the formation of ice crystals but also hinder the evaporation of water molecules from the PAAm gel. As shown in [Supplementary-material supplementary-material-1], a pure water-based hydrogel and a binary solvent-based organohydrogel were placed in an oven at 20°C for 6 h. The volume of the pure hydrogel shrinks to about half of the original one; correspondingly, there is almost no change of the organohydrogel. In order to investigate the effect of glycerol content on the antidrying properties, the as-prepared gels were placed in air and weighed at regular intervals. The weight changes at different times are evaluated in terms of the ratio of *W*_*t*_ (weight at time *t*) to *W*_0_ (weight of the as-prepared gel). As shown in [Supplementary-material supplementary-material-1], the weight retention is significantly dependent on the content of glycerol in gels. After evaporation in air for 8 h, the pure hydrogel has lost 70% of its original weight. Considering that the water content of pure hydrogel is around 90%, after the evaporation process, the hydrogel has lost 80% of its water. With the increase of glycerol content, the weight loss of the gels during evaporation also reduces. Pure glycerol-based organogels even absorb water, resulting in the increase of weight. The organohydrogel with a 1 : 1 ratio of glycerol and water exhibits great stability, and the weight retention after 8 h is about 90%. At relatively higher temperatures (40°C and 60°C), since hydrogel bonds will be weakened at higher temperatures, the evaporation of water molecules accelerates and ordinary hydrogels will lose their functions, but the presence of glycerol can effectively slow the evaporation of water and maintain the shape of the gel ([Supplementary-material supplementary-material-1]). In addition, we found that the introduction of ethylene glycol and sorbitol will also render the gel with antifreezing and antidrying performances. As shown in [Supplementary-material supplementary-material-1], while a 67 wt% glycol solvent organohydrogel maintains its weight in the air, a pure sorbitol-based organogel loses 30% of its weight after 8 h in the air, while the weight retention of the organohydrogel with a 1 : 1 ratio of glycol and water after 8 h is about 80%. Obviously, glycerol shows the best antidrying properties among the three solvents.

### 2.2. Soft Gel Sensor for a Wide Range of Temperatures

**Figure 3:**
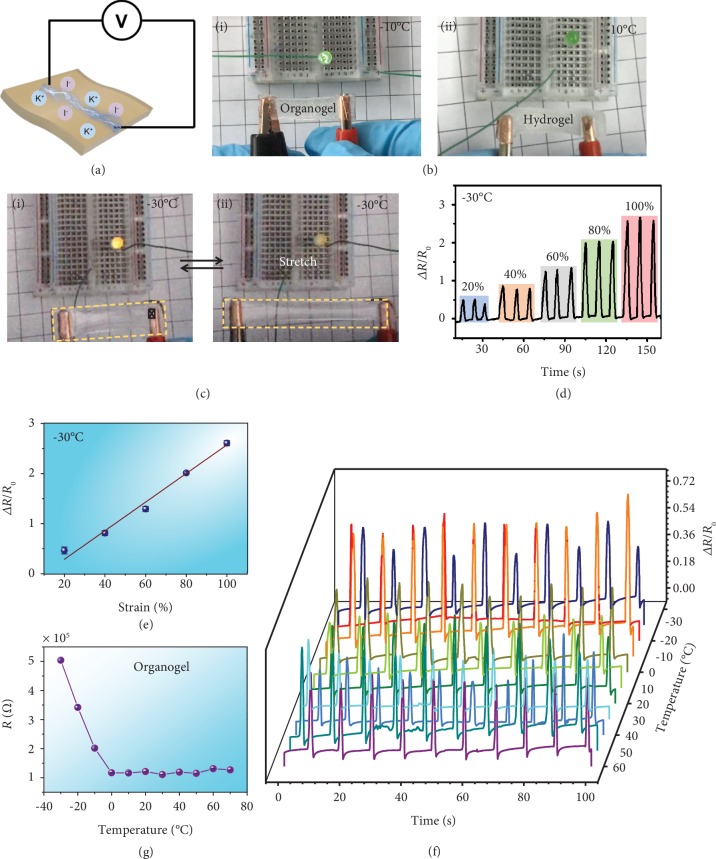
The conductive properties of the KI/glycerol organogel. (a) Schematic diagram of the conductive mechanism of the KI/glycerol PAAm gel. (b) Comparison of conductivity of (i) the organogel and (ii) the hydrogel at -10°C. (c) Control the switch of a light bulb by stretching the organogel at -30°C. (d) Resistance variation curves recorded alternatively for different strains at -30°C. (e) Fitting curve of the resistance change rate under different strains. (f) Resistance variation curves recorded for a 25% strain at different temperatures. (g) Resistance changes of the organogel at different temperatures.

The muscles of animals actuate upon external stimuli, which is usually transmitted through body fluids, and ions in body fluids play an important role in the signal transmittance process. The excellent antifreezing and stretchable behaviors of the gels encouraged us to explore their applications as conductors that could endure extreme temperatures. To enhance the conductivity, potassium iodide (KI) was introduced into PAAm gels. Glycerol breaks the crystal structure of KI, enabling the conductivity of the liquid ([Supplementary-material supplementary-material-1]); thus, the KI/glycerol organogels exhibit good conductivity because of the good solubility of KI in glycerol and the great ion mobility in gels (Figure 3(a)). Figure 3(b) shows the conductive properties of the organogel and ordinary hydrogel at -10°C. The hydrogel freezes and hinders the migration of ions, so the resistance is too large to be used as a conductor and the light bulb is off. On the other side, the bulb could be lit due to the great conductivity and excellent antifreezing properties of the KI/glycerol organogel. In addition, the resistance of the KI/glycerol organogel will change with stretching. As shown in Figure 3(c), when a piece of organogel is connected in a circuit without stretching, the light bulb is turned on, and when the gel is stretched to a 50% strain, the light bulb is extinguished ([Supplementary-material supplementary-material-1], Supporting Information). To further demonstrate the stable conductive behavior of the organogel at subzero temperatures, the influence of the strain on the variation ratio of the electronic resistance (Δ*R*/*R*_0_ = (*R*_0_ − *R*)/*R*_0_) at -30°C was investigated, where *R*_0_ and *R* refer to the resistance without and with strain, respectively. As shown in Figures 3(d) and 3(e), the electric resistance increases when a tensile strain is applied on a conductive gel and it will restore to the original level when the strain is removed. The repeatability of the sensing performance under each strain is very good, and the relationship between the resistance change and the strain can be fitted linearly very well.

The good conductive stability of the gel renders it a great potential as a strain sensor to monitor human physiological motions; as displayed in [Supplementary-material supplementary-material-1], the gel sensor is placed tightly on a finger and the finger bending performance at -30°C could be clearly detected. For many gel-based flexible conductors, the temperature tolerance behavior is usually not satisfactory because antifreezing and antiheating normally cannot be combined in one system. Thanks to the remarkable antifreezing and antidrying properties, the organogel exhibits stable conductive performance at a wide temperature range from -30°C to 60°C (Figure 3(f)), and the electronic resistance decreases with increasing temperature, which is consistent the conductive behavior of KI/glycerol solutions (Figure 3(g) and [Supplementary-material supplementary-material-1]). It can be speculated that as the temperature reduces, the viscosity of the solution would enhance and the ion migration rate in the solution and in the gel would decrease as a result.

### 2.3. Gel Actuator for a Wide Range of Temperatures

**Figure 4:**
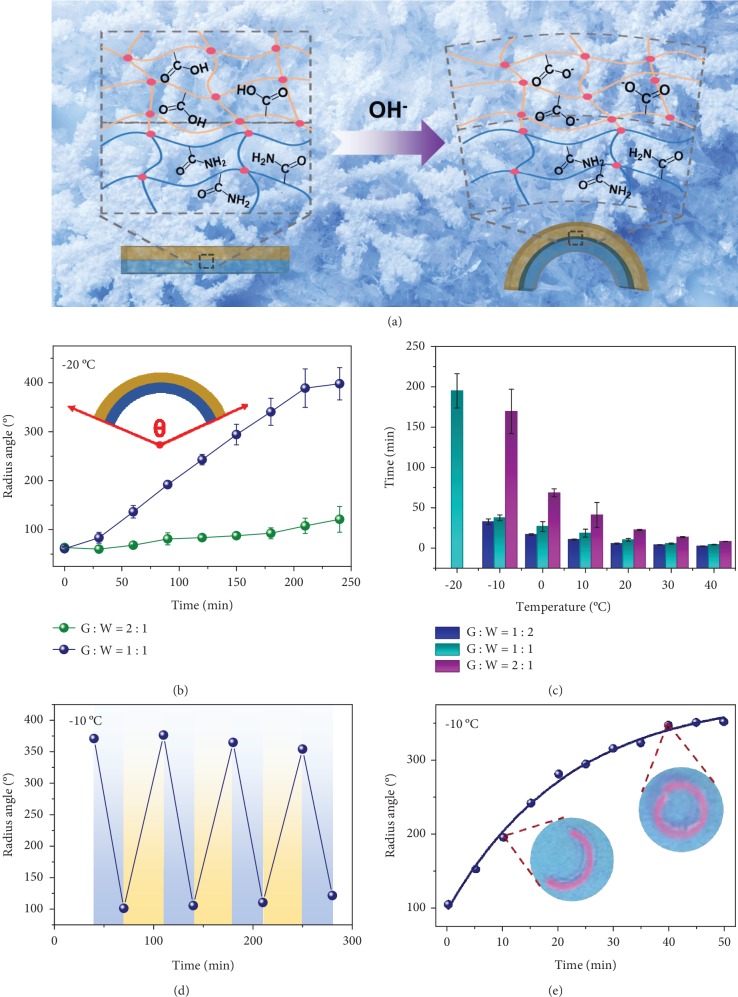
The actuation properties of the bilayer PAAm/PAA organohydrogel. (a) Schematic illustration of deformation of the organohydrogel at subzero temperature. (b) Actuation curves of the bilayer organohydrogel with different solvent components in alkaline solutions at -20°C. (c) The time required for the organohydrogel with different solvent components to reach the 360° deformation angle at different temperatures. (d) Cyclic actuation performances of the bilayer organohydrogel with a solvent composition of 1 : 1 at -10°C. (e) Actuation curves of the bilayer organohydrogel at -10°C after being placed in the air for 7 days.

The outstanding antifreezing performance of the organohydrogel makes it possible to be applied as an actuator that functions at subzero temperatures. Since a PAAm hydrogel normally does not respond to external stimuli in an aqueous environment, in order to construct an actuator that could produce shape transformations, a pH-responsive polyacrylic acid (PAA) layer was introduced as a deformation layer to achieve a bilayer organohydrogel. The PAA layer can be unprotonated under alkaline conditions, and the electrostatic repulsion among the carboxyl groups would cause the PAA layer to swell ([Supplementary-material supplementary-material-1]), and the shape of the bilayer organohydrogel would deform as a result of the swelling of the PAA layer (Figure 4(a)). In order to study the effect of the glycerol content on the actuation performance, a series of mixed solvents with glycerol to water ratios of 2 : 1, 1 : 1, and 1 : 2 were prepared, and the concentration of NaOH was kept at 0.05 M. Since the binary solvent with a ratio of glycerol to water of 1 : 2 would freeze into solid after storing at -20°C for about 1 h, the actuating behaviors of the bilayer organohydrogel at -20°C in the binary solvent with ratios of glycerol to water of 2 : 1 and 1 : 1 were investigated. As shown in Figure 4(b), the deformation rate of the organohydrogel significantly depends on the proportion of water in the solvents; in the solvent of glycerol to water of 2 : 1, the organohydrogel deforms very slowly and no significant changes are observed after 4 h; in the solvent of glycerol to water of 1 : 1, the organohydrogel exhibits a remarkably faster shape deformation. Besides the glycerol content, the shape deformation performance of the organohydrogels is also influenced by temperature. Figure 4(c) and [Supplementary-material supplementary-material-1] display the time required for the organohydrogels to reach a 360° bending angle. With the decrease of temperature, the organohydrogels apparently need more time to deform into a circle. At -20°C, about 200 min is needed for the organohydrogel to bend into a circle in the solvent of glycerol to water of 1 : 1, while this time will reduce to about 8 min when the temperature is increased to 40°C.

At the same temperature, the time is decreased with increasing content of water, which indicates that the presence of more water will benefit the deforming process. Considering the balance between deformation performance and freezing tolerance, a 1 : 1 ratio of glycerol to water was chosen, of which 50 wt% glycerol prevents the gel from freezing, while 50 wt% water ensures an acceptable response rate. At -10°C, the pH-responsive actuating performance of the organohydrogel is proven to be fully reversible (Figure 4(d)); moreover, after being placed in air for 7 days, the organohydrogel still maintains good actuation property at low temperature (Figure 4(e)). Taken together, all the results make the organohydrogel excellent actuators at subzero temperatures.

**Figure 5:**
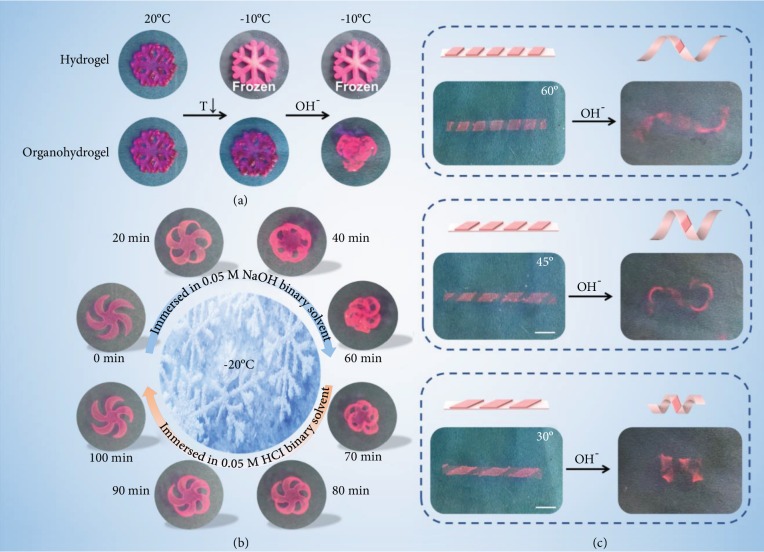
The antifreezing actuation properties of the PAAm/PAA bilayer organohydrogel. (a) The organohydrogel could deform in basic condition at -10°C, while the hydrogel was frozen. (b) Application demonstrations of the bilayer gel through the imitation of flowers. Flower-shaped organohydrogels could produce reversible deformations under acidic and basic conditions. (c) Programmable complex deformations achieved by patterning the design (-10°C). The twist of the gel could be tuned by the angle of the patterned pH-responsive PAA layer.

Not only simple deformation from strip to ring could be realized, complex 3D deformation could also be achieved through advanced programming.  Figure 5(a) shows the great antifreezing performance of the organohydrogel actuator; it could deform in basic solution at -10°C, while the hydrogel is frozen. As shown in Figure 5(b), a predesigned flat organohydrogel, after placing in an alkaline solution, could transform into a three-dimensional flower in about 60 min at -20°C, and the flower could open again if it is transferred into an acid condition. The deformation was used to design a gel actuator that mimics the blossom of a snow lotus ([Supplementary-material supplementary-material-1]). In addition, programmable complex deformations can also be achieved by patterning the design of the pH-responsive layer (Figure 5(c)). By adjusting the angle of the patterned strips, the twist of the gel strip could be controlled.

**Figure 6:**
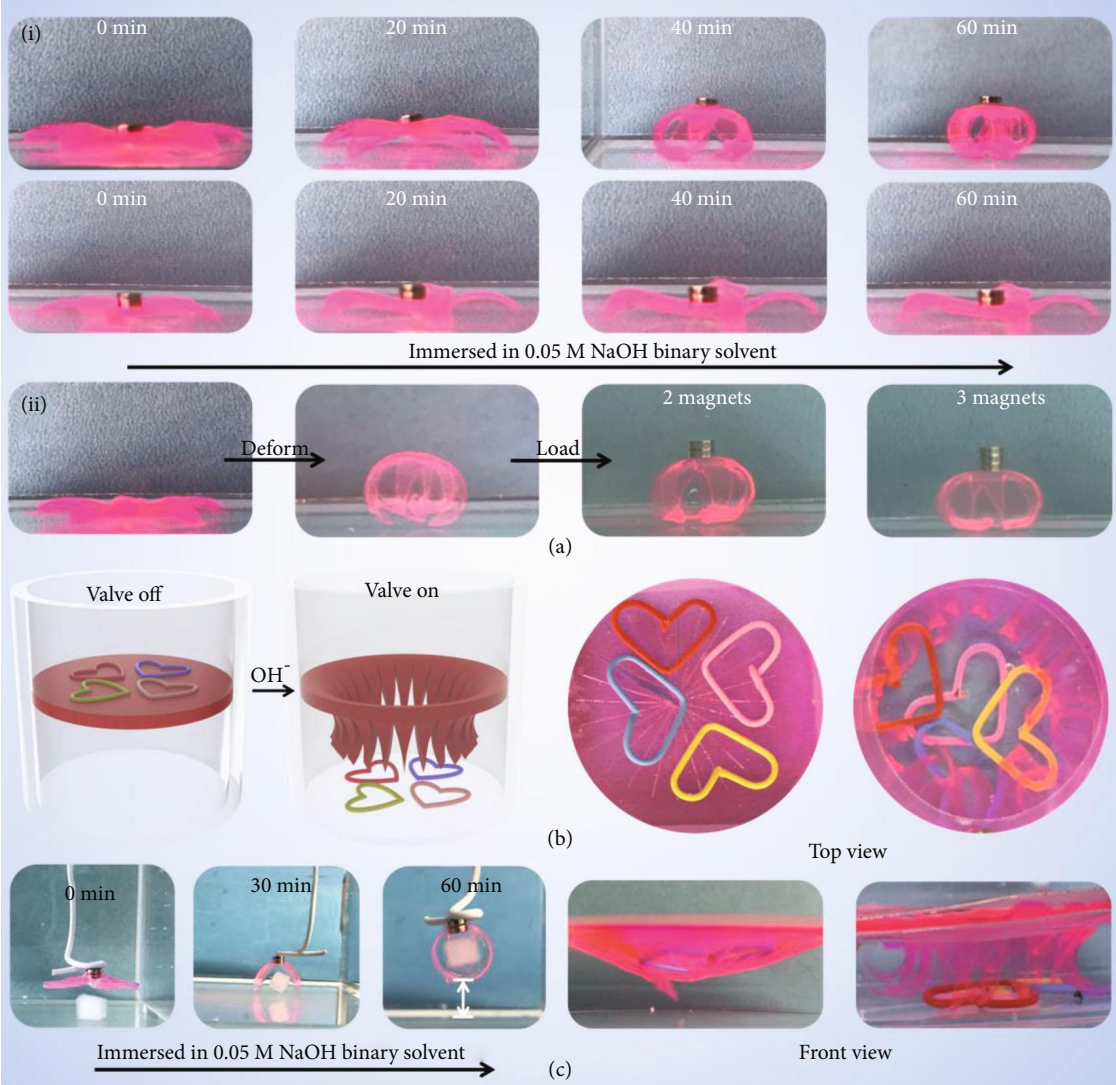
Application demonstrations of the bilayer organohydrogel. (a) The weightlifting robots could lift magnet pieces at -10°C in the alkaline condition (the weight of each magnet is 0.05 g). (b) A circular valve could open in the alkaline condition and liquids/objects could pass through. (c) A robotic arm could grasp an object at -20°C in the alkaline condition.

The outstanding actuating performance suggests the potential of the organohydrogel actuators as mechanical equipment available at subzero temperatures. As shown in Figure 6(a), a weightlifting robot has been designed; it could lift one magnet during the actuating process (-10°C). If two magnets are placed on the top, though its legs show signs of actuating, the weightlifting robot cannot stand up, which is probably because of the small contact area resulting in too much pressure on the center of it. But when the deformation is complete, the weightlifting robot can withstand two or even three magnets. In addition, the maximum load can be tuned by the geometry of the weightlifting robots ([Supplementary-material supplementary-material-1]). Moreover, an artificial valve has been fabricated, and it could open when it is exposed to alkaline condition; liquids/objects could pass through as a result (-10°C, Figure 6(b) and [Supplementary-material supplementary-material-1]). In addition, the organohydrogel actuators could work at an even lower temperature, as shown in Figure 6(c) and [Supplementary-material supplementary-material-1] (Supporting Information); a soft gripper would close slowly and grab the object from the bottom of the water and lift it up at -20°C.

## 3. Discussion

Inspired by the antifreezing behaviors of living creatures, we have developed a bilayer PAAm/PAA asymmetric organohydrogel actuator that can be used at extremely harsh temperatures by introducing glycerol in the system. The strong hydrogen bonds between glycerol and water molecules would prevent the formation of ice crystals at subzero temperatures and endow the organohydrogel with antifreezing abilities. The organohydrogel shows stable mechanical properties below 0°C and could bear various large shape deformations. Moreover, the carboxyl groups of the PAA layer would be unprotonated and drive the bilayer organohydrogel to actuate at extremely low temperatures; organohydrogel flowers, weightlifting robot, artificial valve, and robotic arms have been constructed to demonstrate the freeze-tolerant actuating behavior. In addition, by introducing KI into glycerol, the obtained organogel exhibits a stable sensing performance over a wide range of temperatures from -30°C to 60°C. We hope our strategy will inspire the design and fabrication of novel intelligent materials with extreme temperature tolerance performances.

## 4. Materials and Methods

### 4.1. Materials

Acrylamide (AAm), acrylic acid (AAc), ethylene glycol, glycerol, sorbitol, and potassium iodide were purchased from Shanghai Sinopharm Chemical Reagent Co., Ltd. 2-Hydroxy-4′-(2-hydroxyethoxy)-2-methylpropiophenone (I2959) and N,N′-methylenebis (acrylamide) (BIS) were obtained from Aladdin Shanghai Reagent Co.

### 4.2. Preparation of AAm Hydrogel

AAm monomers (1.2 g), BIS (12 mg), and I2959 (12 mg) were dissolved in 6 mL of deionized water and stirred to obtain a homogenous solution. Then, the solution was transfused to a glass mold, including a layer of silica plate (1 mm) with a specific shape sandwiched between two glass plates (2 mm). The mold was sealed then irradiated under UV light (365 nm) for 4 min.

### 4.3. Preparation of Bilayer Organohydrogel

AAm monomers (1 g), BIS (10 mg), and I2959 (10 mg) were dissolved in 5 mL of a glycerol/water binary solvent and stirred to obtain an AAm pregel solution. AAc monomers (1.0 mL), BIS (10 mg), and I2959 (10 mg) were dissolved in 5 mL of a glycerol/water binary solvent and stirred to obtain an AAc pregel solution. The AAm pregel solution was added into a glass mold including a layer of silica plate (0.5 mm) with a specific shape sandwiched between two glass plates (2 mm). After being irradiated under UV light (365 nm) for 4 min, the top glass piece was removed and another silica plate was placed on the first silica plate. The AAc pregel solution was poured into the second silica plate and sealed with a glass plate. The mold was placed under UV light (365 nm) for 4 min to obtain the bilayer organohydrogel. After polymerization, the gel was removed and placed in glycerol/water solution.

### 4.4. Characterization

The rheological measurements were performed on a Haake MARSIII rheometer equipped with a geometry of 25 mm parallel plates at 25°C. The electrical tests were performed by a CHI 660E electrochemical workstation (CH Instruments, Chenhua Co., Shanghai, China). The tensile and compression tests were performed on a CMT-1104 universal testing machine (CMT-1104, SUST Electrical Equipment Co., Zhuhai, China). The dynamic mechanical analysis was performed on a DMAQ800 analyzer (DMAQ800, TA, America). The samples were frozen in liquid nitrogen for 10 min before lyophilizing with a freeze drier (FD-1C-50, Beijing Boyikang) at −35°C for about 48 h.

### 4.5. Evaluation of Actuation Behaviors

A straight strip of bilayer gels with size of 20 mm × 2 mm × 1 mm was immersed into the same oil/water solution they contained as the solvent to achieve a swelling balance. Then, they were immersed in a 0.05 M NaOH solution with different oil/water ratios. The degree of actuation was determined by the central angle *θ*.

### 4.6. Preparation of the Conductive Organogel

Potassium iodide (40 g) was dissolved in 200 mL of glycerol to obtain the KI/glycerol solution. Then, the as-prepared PAAm hydrogel was soaked in the KI/glycerol solution for 24 h to achieve sufficient solvent exchange.

### 4.7. Evaluation of Conductivity Behaviors

A rectangular piece of KI/glycerol organogels with a size of 40 mm × 10 mm × 1 mm was connected between two electrodes and was added a fixed voltage of 1 V. Then, the gel block was subjected to a cyclic tensile test under a 25% strain at different temperatures.

## Supplementary Materials

Supplementary 1 Figure S1: mechanical properties of the gel at low temperatures. Figure S2: the antidrying properties of the PAAm organohydrogels. Figure S3: mass change curves of organohydrogels with other oil solvents. Figure S4: photos of using the conductive organogel to illuminate the light bulb. Figure S5: resistance changes of KI/glycerol solvents at different temperatures. Figure S6: the organogel as a wearable sensor to monitor finger bending at -30°C. Figure S7: swelling change of PAA and PAAm organohydrogel under the alkaline condition. Figure S8: actuation curves of the bilayer organohydrogel with different solvent components. Figure S9: application demonstrations of our bilayer gel in imitating the blossom of snow lotuses. Figure S10: loading performance of weightlifting robots with different geometries. Figure S11: application demonstrations of the bilayer gel as an artificial valve at -10°C.

Supplementary 2 Movie S1: bulb switch controlled by stretching the organogel.

Supplementary 3 Movie S2: robotic arms working at subzero temperature.
