# Supplementary material for: Antifreezing and Stretchable Organohydrogels as Soft Actuators
Source: Research (Wash D C). 2019 Dec 13;2019:2384347. doi: 10.34133/2019/2384347 (PMC6944494; doi:10.34133/2019/2384347)
Supplement: Supplementary 1 — Figure S1: mechanical properties of the gel at low temperatures. Figure S2: the antidrying properties of the PAAm organohydrogels. Figure S3: mass change curves of organohydrogels with other oil solvents. Figure S4: photos of using the conductive organogel to illuminate the light bulb. Figure S5: resistance changes of KI/glycerol solvents at different temperatures. Figure S6: the organogel as a wearable sensor to monitor finger bending at -30°C. Figure S7: swelling change of PAA and PAAm organohydrogel under the alkaline condition. Figure S8: actuation curves of the bilayer organohydrogel with different solvent components. Figure S9: application demonstrations of our bilayer gel in imitating the blossom of snow lotuses. Figure S10: loading performance of weightlifting robots with different geometries. Figure S11: application demonstrations of the bilayer gel as an artificial valve at -10°C. [file 2384347.f1.docx]

**Supplementary Materials**

**Anti-freezing Organohydrogels as Soft Actuators**

Yukun Jian^1,2^, Baoyi Wu^1^, Xiaoxia Le^1,2^, Yun Liang^1,2^, Yuchong Zhang^1,2^, Dachuan Zhang^1^, Ling Zhang^1,2^, Wei Lu^1,2^, Jiawei Zhang^1,2^*, Tao Chen^1,2^*

^1^ Key Laboratory of Marine Materials and Related Technologies, Zhejiang Key Laboratory of Marine Materials and Protective Technologies, Ningbo Institute of Material Technology and Engineering, Chinese Academy of Sciences, Ningbo, 315201, China

^2^ University of Chinese Academy of Sciences, 19A Yuquan Road, Beijing 100049, China

Correspondence should be addressed to Jiawei Zhang and Tao Chen. zhangjiawei@nimte.ac.cn, [tao.chen@nimte.ac.cn](mailto:tao.chen@nimte.ac.cn)

**Table of Contents**

**Figure S1** Mechanical properties of gel at low temperatures.  **3**

**Figure S2** The anti-drying properties of the PAAm organohydrogels.  **3**

**Figure S3** Mass change curves of organohydrogels with other oil solvents.  **4**

**Figure S4** Photos of using the conductive organogel to illuminate the light bulb.  **4**

**Figure S5** Resistance changes of KI/Glycerol solvents at different temperatures. **5**

**Figure S6** The organogel as a wearable sensor to monitor finger bending at -30 ℃.  **5**

**Figure S7** Swelling change of PAA and PAAm organohydrogel under alkaline condition.  **6**

**Figure S8** Actuation curves of the bilayer organohydrogel with different solvent components.  **6**

**Figure S9** Application demonstrations of our bilayer gel to imitating the blossom of snow lotus. **7**

**Figure S10** Loading performance of weightlifting robots with different geometries. **7**

**Figure S11** Application demonstrations of the bilayer gel as artificial valve at -10 ℃. **8**

**Movie S1.** Bulb switch controlled by stretching the organogel. **8**

**Movie S2.** Robotic arms worked at subzero temperature. **8**


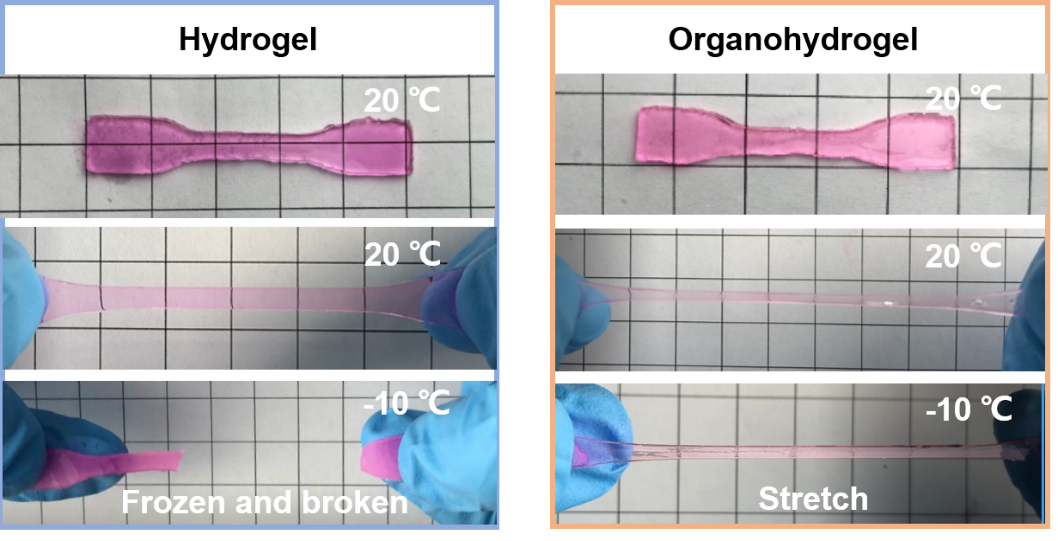


**Figure S1.** Hydrogel and organohydrogel are stretched at 20 ℃ and -10 ℃, respectively.

**
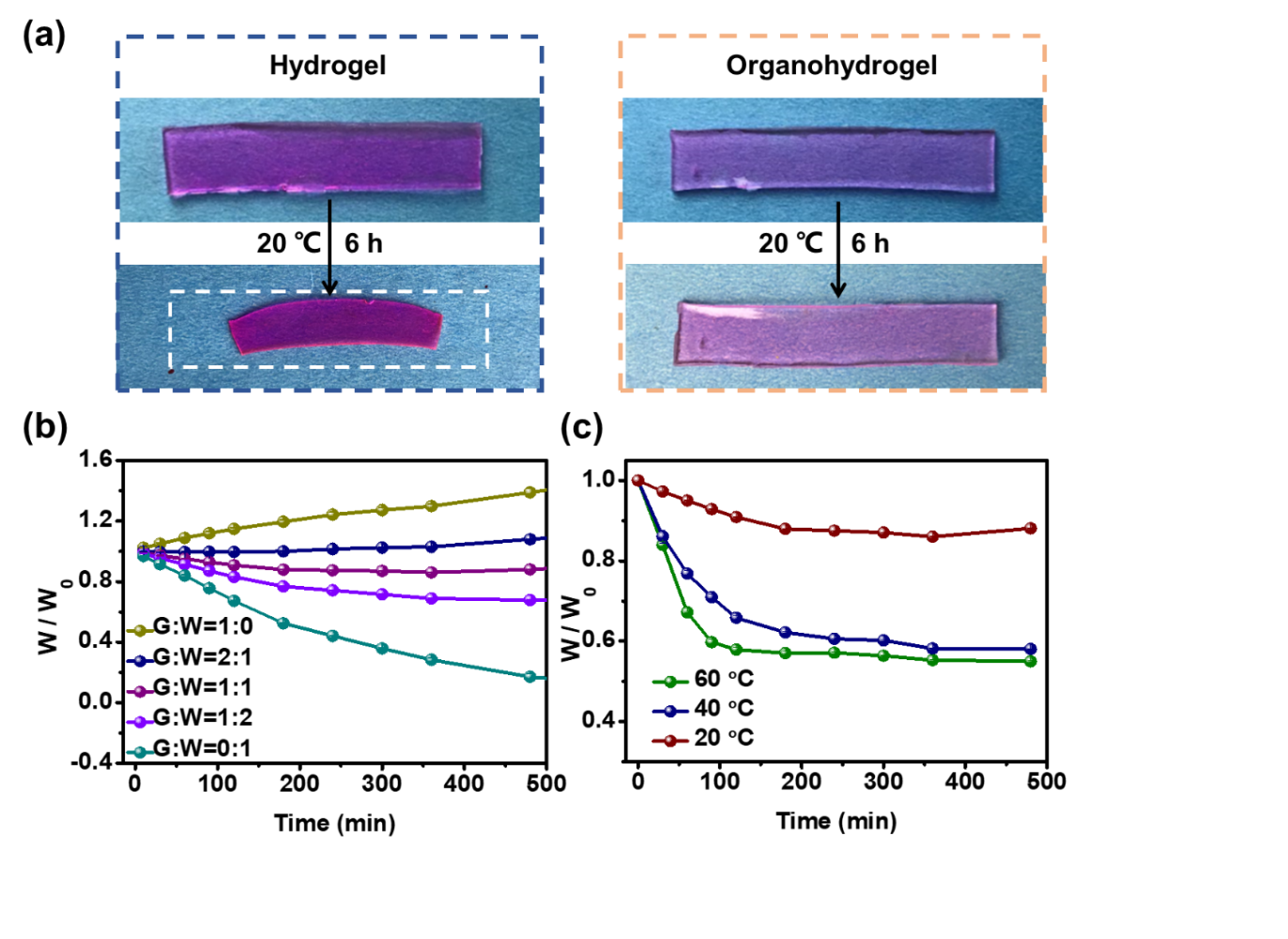
**

**Figure S2.** The anti-drying properties of the PAAm organohydrogels. (a) Digital photos of hydrogel and organohydrogel with a 1:1 ratio of glycerol and water before and after being placed at 20 ℃ for 6 h, respectively. (b) Mass change curves of organohydrogel with different solvent components in air at room temperature. (c) Mass change curves of organohydrogel with a 1:1 ratio of glycerol and water at 20 ℃, 40 ℃, and 60 ℃, respectively.


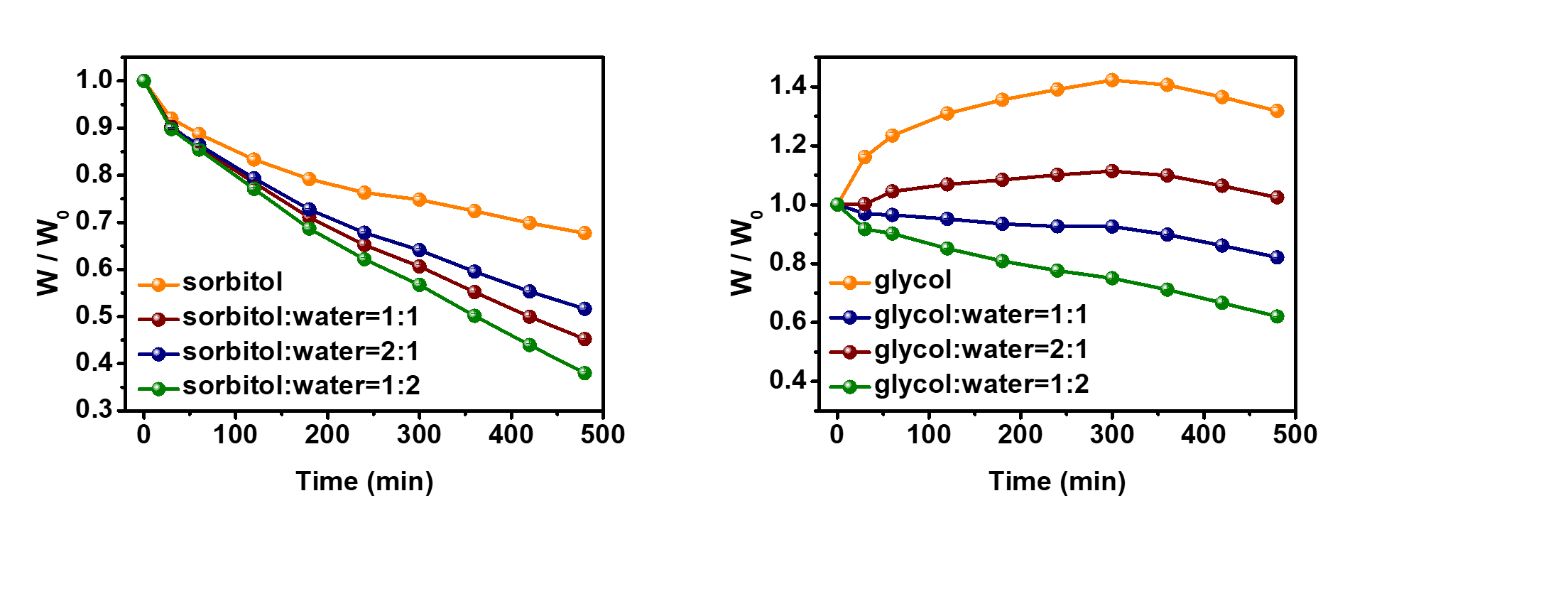


**Figure S3**. Mass change curves of organohydrogels with sorbitol/water solvents and glycol/water solvents in air at room temperature.


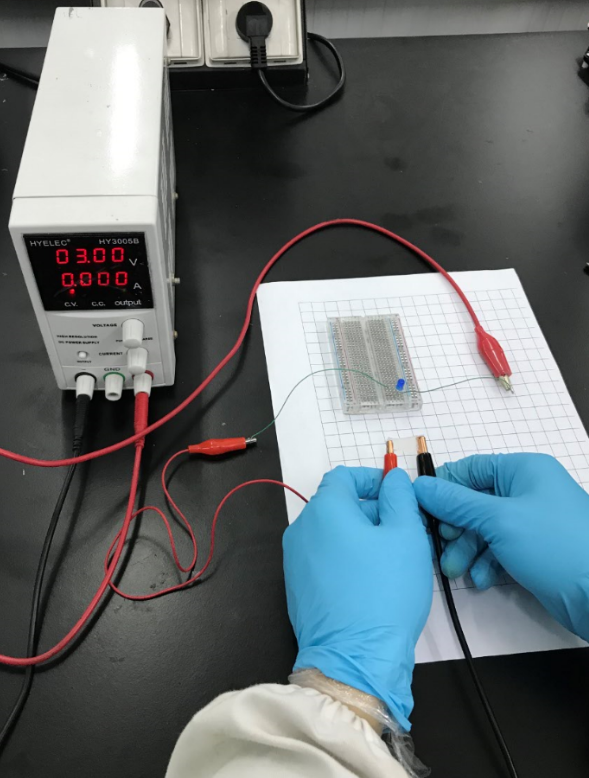


**Figure S4.** Photos of using the conductive organogel to illuminate the light bulb.


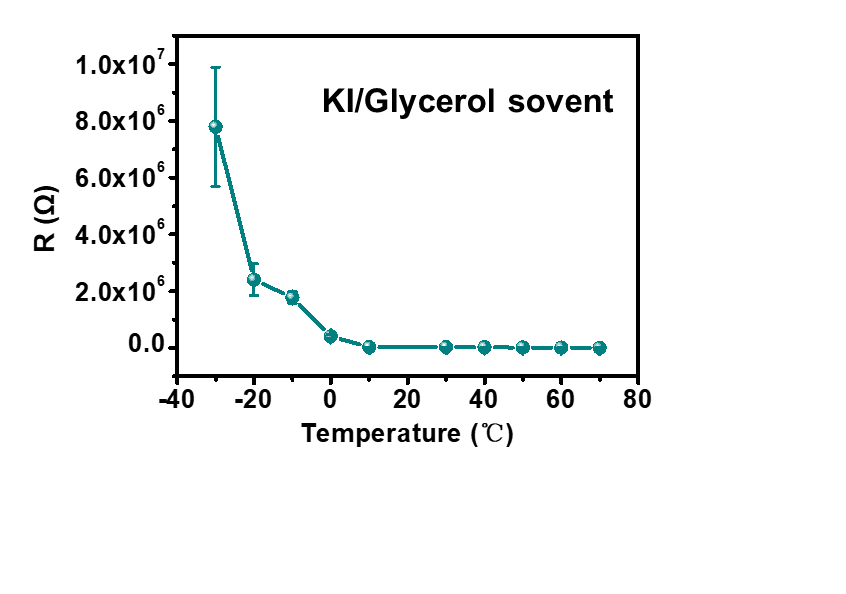


**Figure S5.** Resistance changes of KI/Glycerol solvents at different temperatures.


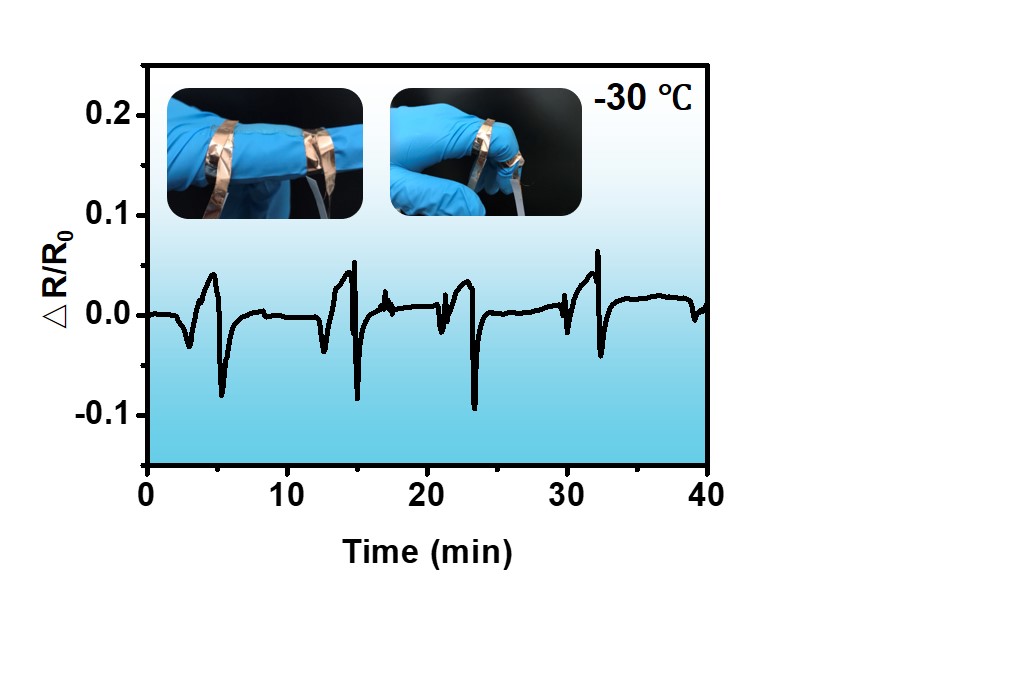


**Figure S6**. The organogel as a wearable sensor to monitor finger bending at -30 ℃. Inset: photographs of finger motions.


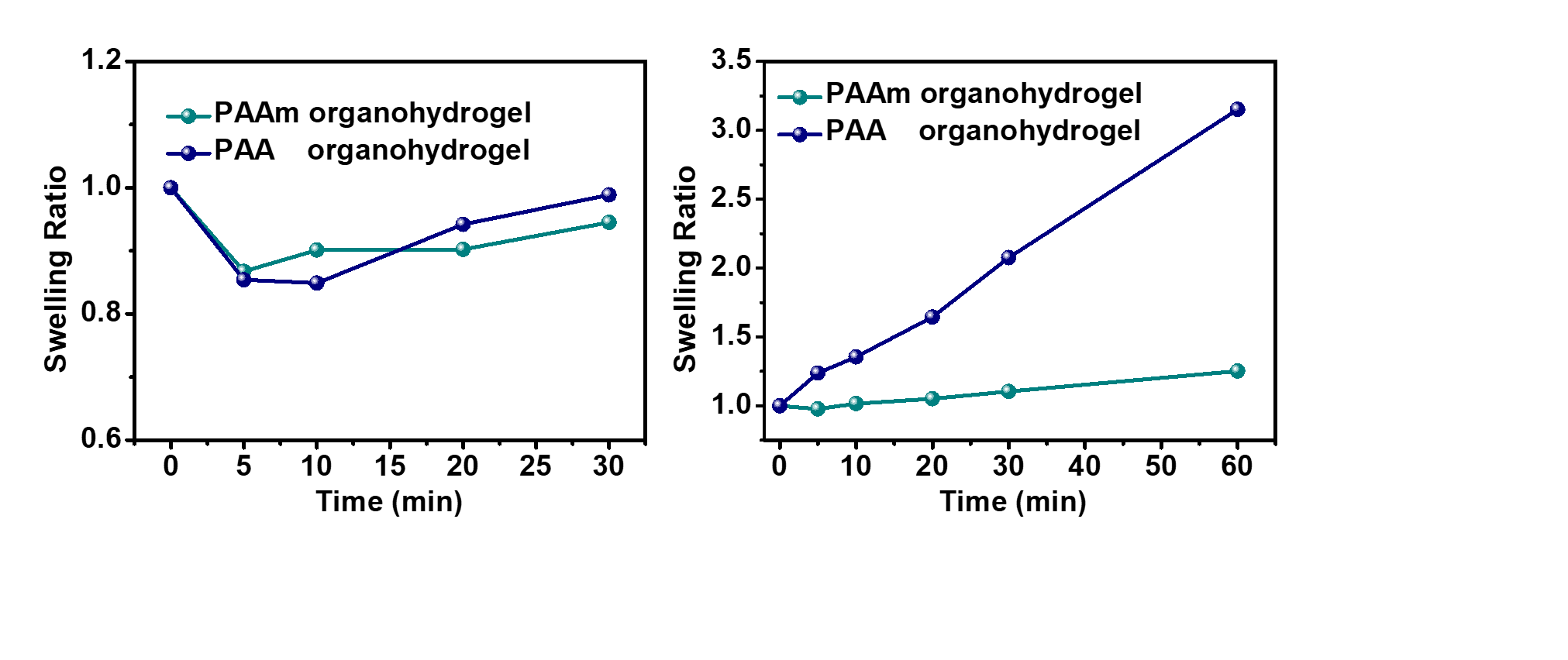


**Figure S7.** Swelling change of PAA and PAAm organohydrogel under alkaline condition.


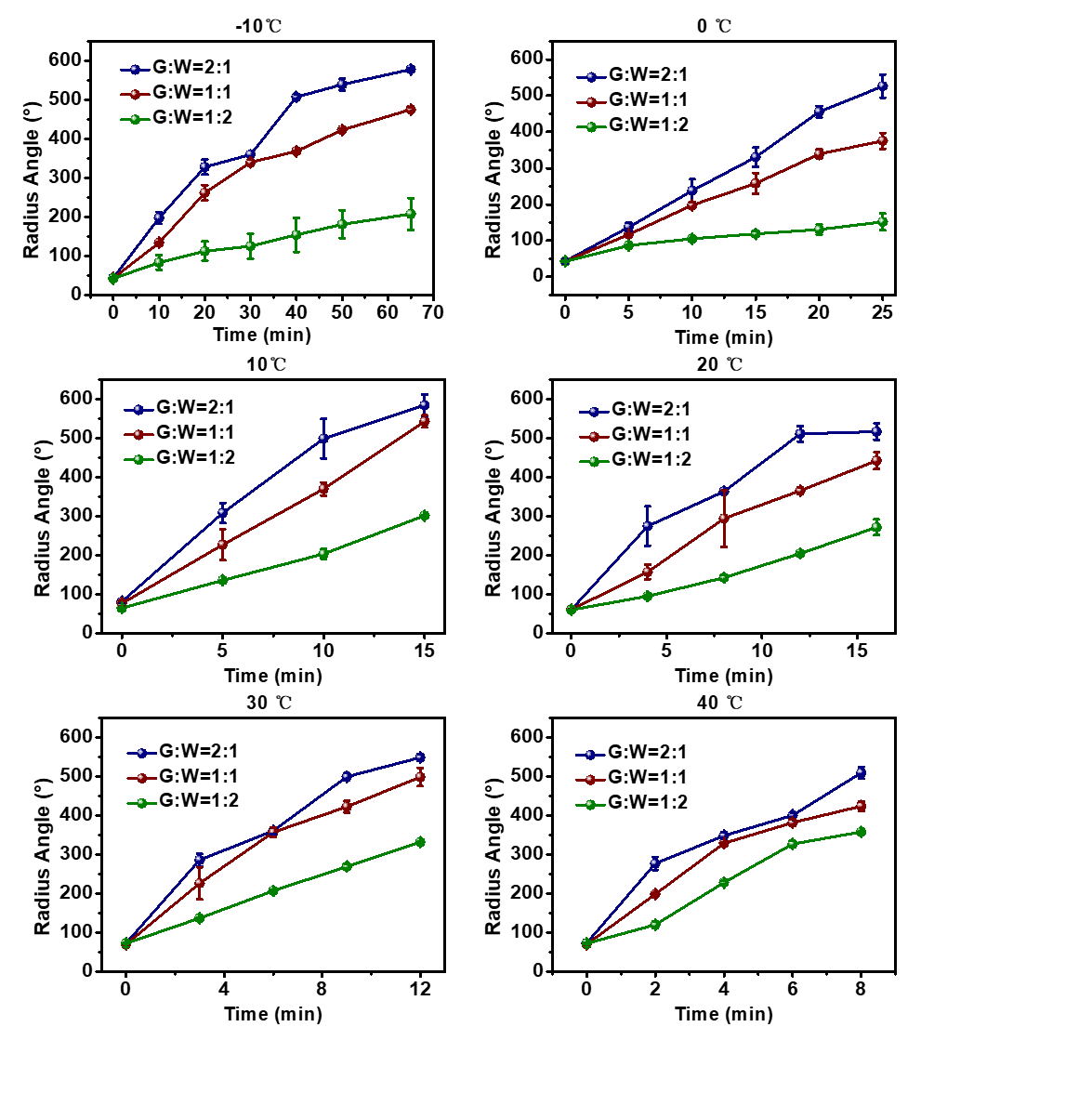


**Figure S8.** Actuation curves of the bilayer organohydrogel with different solvent components in alkaline solutions at -10~40 ℃.

**
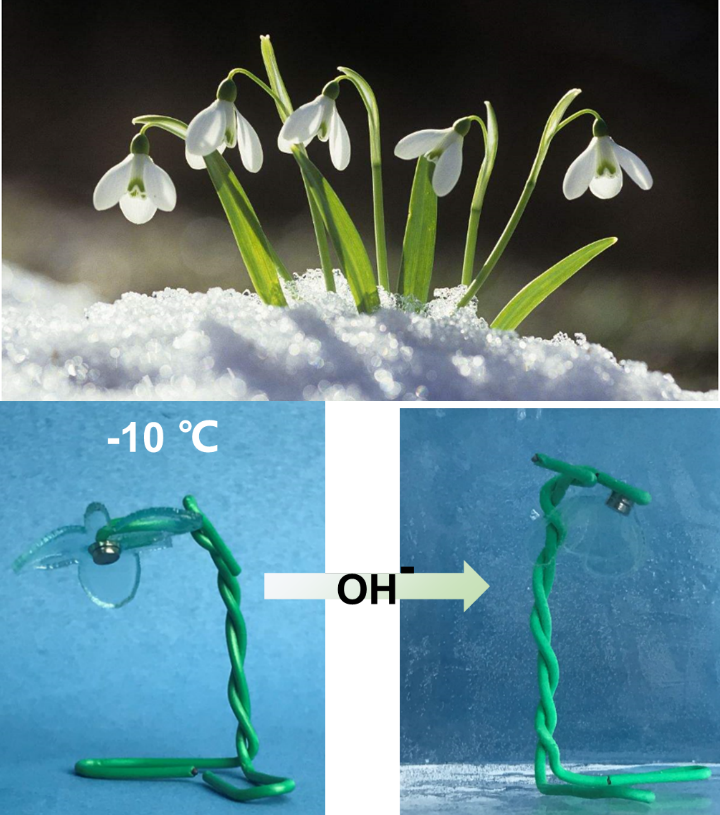
**

**Figure S9.** Application demonstrations of our bilayer gel to imitating the blossom of snow lotus at -10 ℃.


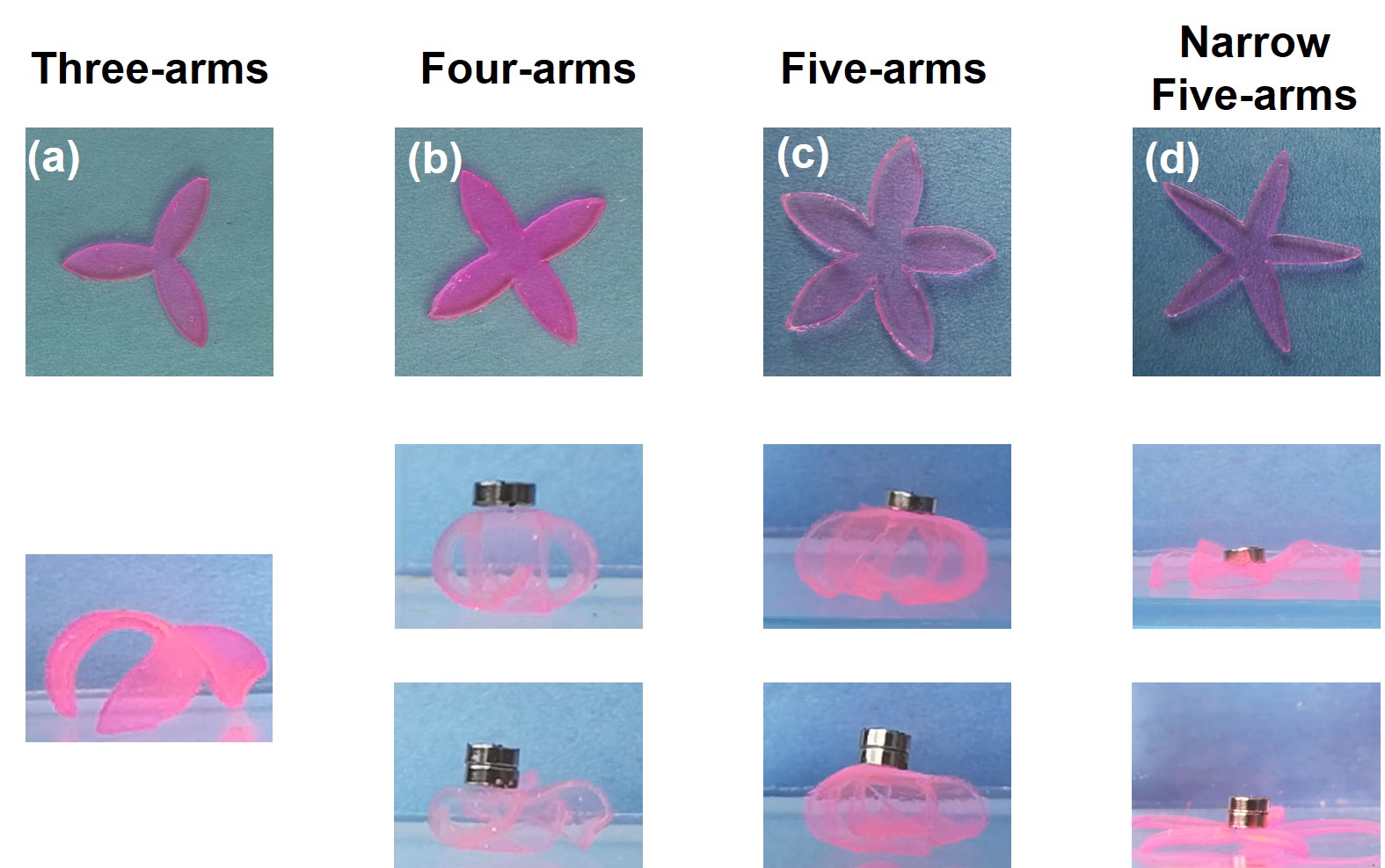


**Figure S10.** Loading performance of weightlifting robots with different geometries (The weight of each magnet is 0.25 g).


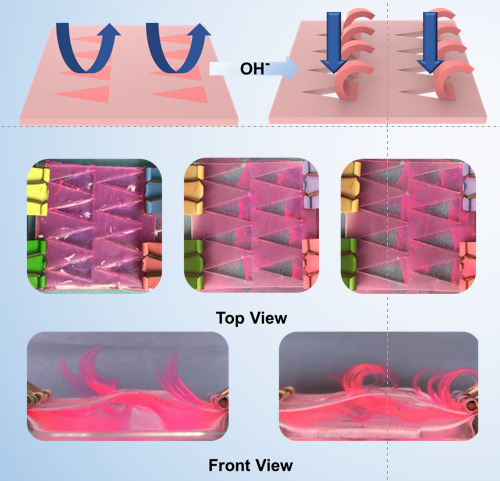


**Figure S11.** Application demonstrations of the bilayer gel as artificial valve at -10 ℃.

**Movie S1.** Bulb switch controlled by stretching the organogel.

**Movie S2.** Robotic arms worked at subzero temperature.
